# Availability and stability of palliative care for family members of terminally ill patients in an integrated model of health and social care

**DOI:** 10.1186/s12904-024-01475-7

**Published:** 2024-06-05

**Authors:** Chunyan Wang, Shaojie Bi, Yanxia Lu, Yuli Li, Bing Han, Min Xu, Guiyue Meng, Qingbo Zhou

**Affiliations:** 1 Department of Geriatrics, The Second Hospital, Cheeloo College of Medicine, Shandong University, Jinan, 250033 China; 2 Department of Cardiology, The Second Hospital, Cheeloo College of Medicine, Shandong University, Jinan, 250033 China; 3 Department of Medical Psychology and Ethics, School of Basic Medical Sciences, Cheeloo College of Medicine, Shandong University, Jinan, 250012 China; 4 School of Nursing and Rehabilitation, Cheeloo College of Medicine, Shandong University, Jinan, 250012 China; 5 Department of Neurology, The Second Hospital, Cheeloo College of Medicine, Shandong University, Jinan, China

**Keywords:** Palliative care, Quality of life, Family burden, Psychological impact, Health and social care integration

## Abstract

**Background:**

Palliative care and the integration of health and social care have gradually become the key direction of development to address the aging of the population and the growing burden of multimorbidity at the end of life in the elderly.

**Aims:**

To explore the benefits/effectiveness of the availability and stability of palliative care for family members of terminally ill patients in an integrated institution for health and social care.

**Methods:**

This prospective observational study was conducted at an integrated institution for health and social care. 230 patients with terminal illness who received palliative care and their family members were included. Questionnaires and scales were administered to the family members of patients during the palliative care process, including quality-of-life (SF-8), family burden (FBSD, CBI), anxiety (HAMA), and distress (DT). We used paired t-tests and correlation analyses to analyze the data pertaining to our research questions.

**Results:**

In the integrated institution for health and social care, palliative care can effectively improve quality of life, reduce the family’s burden and relieve psychological impact for family members of terminally ill patients. Palliative care was an independent influencing factor on the quality of life, family burden, and psychosocial status. Independently of patient-related and family-related factors, the results are stable and widely applicable.

**Conclusion:**

The findings underline the availability and stability of palliative care and the popularization of an integrated service model of health and social care for elder adults.

## Introduction

Population aging is a serious problem gradually emerging in China and even in the world [1]. Due to the large population base and the rapid acceleration of population aging, China has been in an aging society for a long time [2, 3]. The grim reality of dying with multimorbidity makes palliative care for the elderly widely concerned. Ensuring the elderly die with dignity, avoiding ineffective rescue and reducing excessive medical treatment, and lightening the burden on families and society, has become an urgent problem to be solved [4, 5].

Under the severe demand of ageing populations and elderly care needs, governments around the world are seeking to integrate health and social care systems in an attempt to meet the triple aim of improving the individual experience of care, improving the health of populations, and reducing costs. The conclusion is that to form a systems-wide approach to health and social care integration to go beyond local and professional level change, which is necessary to meet the complex and growing needs of an ageing, multimorbid population [6, 7]. The integrated model of health and social care delivery encompasses a coordinated approach to providing comprehensive services to individuals with complex needs. It integrates various aspects of health care, personal care, and supportive services to address the needs of vulnerable populations, particularly the elderly and those with limited self-care abilities, with the goal of improving and maintaining their health status and quality of life [8–12]. Since its inception, this model has aimed to bridge the gap between fragmented care systems and ensure seamless delivery of services across different care settings.

Since 2016, the Service Model of Health and Social Care Integration for Elder Adults has been applied in China. By deeply integrating the functions of medical treatment and elderly care, it solves the most basic living needs and physical and mental health needs of the elderly [13, 14]. A service system integrating daily care, health examination, disease diagnosis, rehabilitation and palliative care has been formed. Since its development in recent years, this integrated model has become a key direction of development. The advantages beyond traditional elderly care and medical care have begun to be highlighted [15, 16].

However, the daily care and medical treatment module still cannot meet the basic needs. Especially for the elderly at the end of life, painful disease symptoms, surgery, and cardiopulmonary resuscitation are experienced against their own will. Even after losing consciousness, they remain subject to a variety of life-sustaining medical equipment and continue to receive traumatic treatment until the last moment of life, which makes the dying process painful [17, 18]. At the same time, the family members also experience substantial burdens, including poor quality of life, heavy burdens of care, and psychological impact [19, 20]. With the development of society, the requirement for the quality of life and the understanding of death continues to improve. Improving the quality of life of elders in the final stage of life and reducing the burden of families have been paid increasing attention.

Given the evolving societal expectations and understanding of death, there is a growing emphasis on enhancing the quality of life for elders in their final stage and alleviating family burdens. Thus, the integration of health and social care institutions strives to establish a comprehensive service system encompassing daily care, medical treatment, and palliative care. The consensus-based definition of palliative care in 2020 is as follows [21]: palliative care is the active holistic care of individuals across all ages with serious health-related suffering due to severe illness and especially of those near the end of life. The aim is to improve the quality of life of patients, their families, and their caregivers. Palliative care provides a comprehensive medical care practice for patients with incurable terminal chronic diseases or serious diseases and their families, following their wishes, and through early identification, comprehensive assessment and effective control of symptoms, to prevent and relieve the physical, psychological, social and spiritual suffering; obtain information; and make choices, thereby improve the quality of life of patients and their families. It does not accelerate or delay the process of death, regarding death as a normal life process and providing active services for patients to make them as comfortable and dignified as possible during the end of life [22]. At the same time, it provides supporting help for family members to reduce the burden of care and psychological impact and to gradually accept the process of patients from life to death [23, 24]. Palliative care, as an important part of the elderly health service system and the last link of medical and health services, is of great significance to relieve the pain of disease and maintain the dignity of life for both patients and their families [25, 26].

Despite the considerable progress in palliative care research, there remains a notable gap in understanding its impact on family members of terminally ill patients, especially within integrated health and social care institutions. While previous studies have emphasized the positive effects of palliative care on patient outcomes, less attention has been paid to its influence on family members’ well-being. Therefore, this study aims to address this gap by specifically investigating the influence of palliative care on family members within integrated health and social care settings, providing valuable insights into the effectiveness and benefits of these services for families facing end-of-life challenges. By elucidating the role of palliative care in enhancing family well-being, this research seeks to inform future palliative care development efforts and policy initiatives aimed at improving end-of-life care for elderly individuals and their families.

Furthermore, a significant gap persists between the need for and access to these services. According to WHO, many individuals with life-threatening illnesses do not receive the palliative care services they require [27, 28]. In China, terminally ill patients often experience lower quality of life, reflecting the substantial disparity between the demand and availability of palliative care services [29]. Therefore, the research aim of our study was to clarify the benefits/effectiveness of the availability and stability of palliative care by examining the influence of palliative care on family members of terminally ill patients in the integrated institution for health and social care, thus providing a basis for the further development of palliative care.

## Methods

### The sample

This study included a consecutive sample of family members of patients with terminal illness receiving palliative care for over a month at the Geriatric department of the Southern Branch of the Second Hospital of Shandong University (Jinan Shande Nursing Home), one of the first integrated health and social care institution in Shandong province, China. The data collection period spanned from January 2018 to December 2021. Inclusion criteria involved the primary family member with biological or social relationship, aged 18 or above, with normal cognitive function and behavioral ability. Exclusion criteria comprised individuals receiving palliative care for less than a month, those transferred to other medical institutions unavailable for study completion, and cases where family members declined participation.

### Palliative care mode

Palliative care constitutes a systematic approach provided to patients with terminal illnesses and their families, aiming to enhance their quality of life and their ability to cope with crises. It was delivered by an interdisciplinary team comprising doctors, nurses, and caregivers, collaborating to address the physical, psychological, social, and spiritual needs of patients and their families. Importantly, palliative care acknowledges death as a natural part of life cycle, neither speeding up nor delaying it. Instead, it emphasizes early recognition of symptoms and suffering, rigorous assessment, and effective management to address the physical, psychological, social and spiritual needs of both patients and their families [21, 30]. Specifically, palliative care services included symptom management, psychosocial support, communication and decision-making assistance, advance care planning, coordination of care, spiritual care, and bereavement support. Symptom management involved effectively alleviating pain, shortness of breath, nausea, and fatigue. Psychosocial support included counseling, psychotherapy, and support groups to address anxiety, depression, grief, and existential distress. Communication and decision-making assistance facilitated open discussions about treatment options, goals of care, and end-of-life preferences. Advance care planning helped patients document their preferences for medical treatment and end-of-life care. Coordination of care ensured holistic care delivery by collaborating with other healthcare providers. Spiritual care addressed spiritual and existential concerns, while bereavement support extended counseling and resources to families coping with grief and loss.

The patient-centric elements of the palliative care system in this study encompassed concern and care for patients, clean and comfortable environment, symptom control (such as pain, breathing difficulty, fidgetiness), and the ability and team-cooperation of the doctors, nurses and caregivers. The family-centric elements of the palliative care system included adjusting care to the evolving needs of patients’ families, including acknowledging and providing reassurance over the role as family member, assisting in navigating the healthcare system, acknowledging and exploring patient’s condition, providing information and advice, aiding in decision-making, maintaining continuity in nurse–family member relationships, monitoring family members’ well-being, listening, enabling the expression of emotions, offering bereavement support, and providing benefits [31–33]. These palliative care services were integral components of the study’s methodology, aimed at assessing the impact of palliative care on family members of terminally ill patients within an integrated institution for health and social care.

### Study design

This prospective study was conducted at the Geriatric department of the Southern Branch of the Second Hospital of Shandong University (Jinan Shande Nursing Home), Shandong, China. General information included hospitalization and nursing care related variables, and communication with participants. Research data were collected by questionnaires and validated scales from family members before admission to palliative care and through the experience of palliative care respectively. According to the reference guidance [33], we chose 1 month as the evaluation time of palliative care, for the results were relatively stable from that. Informed consent was obtained from all participating family members. The study protocol received approval from the Medical Ethics Committee of the Second Hospital of Shandong University [No. KYLL-2021(KJ)P-0478].

### Measurements of outcomes

In order to assess the effect of palliative care in the integrated institution for health and social care, a series of questionnaires and validated scales were administered by study staff to patients’ family members, including Short Form-8 Health Survey (SF-8), Family Burden Scale of Diseases (FBSD), Caregiver Burden Inventory (CBI), Hamilton Anxiety Scale (HAMA), Distress Thermometer (DT) and an adapted problem list [20, 24, 34].

### Statistical analysis

Continuous data were tested for normality. The data conforming to normal distribution were represented by mean and standard deviation (Mean ± SD). Otherwise, they were expressed by median and quartile spacing (Median, IQR). Classified data were expressed as case number and percentage. Descriptive analysis was used to examine sample characteristics and patient and family member outcomes regarding quality of life, family burden and psychological disorder. Differences before and after palliative care were determined using a paired *t*-test. The *P-*value of less than 0.05 was considered statistically different. Univariate and multivariate regression analysis were conducted to study the influencing factors of patients and family members, and the role of palliative care on quality of life, family burden and psychosocial status for family members. Regression coefficients and 95% confidence intervals (95%CI) were used to indicate statistical significance. All the analyses were performed with the statistical software packages R 4.1.3 (http://www.R-project.org accessed on 10 March 2022, The R Foundation) and Free Statistics software versions 1.7.

## Results

### Patient and family member characteristics

According to the inclusion and exclusion criteria, 230 patients and their family members were included in this study. 182 of the patients died and 48 of them were still under palliative care. Patient and family member characteristics were recorded at enrollment. The mean age of patients was 81.61 years (SD 8.94; range 52–98), and 66.09% were male. Patients were admitted from other hospital (36% of the cases), specialized nursing homes (18%), and home care with nursing service (46%), with 44.78% of the patients treated with invasive treatment previously. Performance status (ECOG score) was ≤ 2 in 1.30%, 3 in 25.22%, 4 in 73.48%. Conscious state was awake in 38.74%, somnolence in 20.87%, confusion in 26.96%, and coma in 10.43%. Nutritional risk (NRS2002) was ≥ 3 in 85.22% of the patients. Among the major diagnosis of patients, cardiovascular and cerebrovascular diseases ranked first (50.87%), followed by cancer (22.61%), infectious disease (19.13%), trauma (4.35%), and autoimmune disease (3.04%). The mean age of the participating family members was 56.25 (SD 9.79; range 30–74), and 53.04% of the sample was male. Most of them had caring experience for patients. The mean length of the caregiving period was 22.87 month (SD 35.11; range 0.2–122) and the daily time spent on caregiving was 9.25 h (SD 7.86; range 3–24 h). All the details of patient and family member characteristics are recorded in Table 1.

**Table 1:** Characteristics of patients and families members (*N* = 230)

Characteristics of patients		*n*	%
Gender	Male	152	66.09
	Female	78	33.91
Age (years), Mean ± SD	81.57 ± 8.94		
	18–39	0	0.00
	40–59	6	2.61
	60–79	72	31.30
	> 80	152	66.09
Religion	Yes	12	5.21
	No	218	94.78
Marital status	Single	3	1.30
	Married	18	7.83
	Divorced/ Widowed	209	90.87
Education	≤Junior high school	86	37.39
	High school	79	34.35
	College	54	23.48
	≥Graduate	11	4.78
Occupation	Employed	0	0.00
	Unemployed	47	20.43
	Retirement	183	79.57
Medical insurance	Social insurance	207	90.00
	Commercial insurance	12	5.22
	Self-paying	11	4.78
Major diagnosis	Cancer	52	22.61
	Cerebrovascular disease	117	50.87
	Infectious disease	44	19.13
	Autoimmune disease	7	3.04
	Trauma	10	4.35
Conscious state	Awake	96	38.74
	Somnolence	48	20.87
	Confusion	62	26.96
	Coma	24	10.43
Performance status	ECOG ^a^ ≤2	3	1.30
	ECOG = 3	58	25.22
	ECOG = 4	169	73.48
Nutritional risk	NRS2002 < 3	34	14.78
	NRS2002 ≥ 3	196	85.22
Invasive experience	Yes	103	44.78
	No	127	55.22
Time1 ^b^ (months), Median (IQR)	9.67 (2.17, 36.50)		
	< 12 months	120	52.17
	≥ 12 months	110	47.83
Time2 ^c^ (months), Median (IQR)	7.57 (2.37, 13.33)		
	< 12 months	160	69.57
	≥ 12 months	70	30.43
Expense ^d^ (yuan/month)	< 5000	95	41.30
	5000–10,000	38	16.52
	> 10,000	97	42.18
**Characteristics of family members**		**n**	**%**
Gender	Male	122	53.04
	Female	108	46.96
Age (years), Mean ± SD	56.25 ± 9.80		
	18–39	17	7.39
	40–59	88	38.26
	60–79	125	54.35
	> 80	0	0.00
Religion	Yes	7	3.04
	No	223	96.96
Marital status	Single	3	1.30
	Married	221	96.09
	Divorced/ Widowed	6	2.61
Education	≤Junior high school	0	0.00
	High school	78	33.91
	College	103	44.78
	≥Graduate	49	21.30
Occupation	Employed	92	40.00
	Unemployed	6	2.61
	Retirement	132	57.39
Relationship to patient	Spouse	13	5.65
	Child	215	93.48
	Sibling	2	0.87

^a^ ECOG: Eastern Cooperative Oncology Group

^b^ Time1: the time from disease onset to palliative care

^c^ Time2: the duration of palliative care

^d^ Expense: monthly expenses for patient

### Quality of life

The quality of life of family members was tested using the Short Form-8 Health Survey (SF-8) before admission to palliative care and 1 month through the experience of palliative care. The scale consists of eight items, divided into physical and psychological dimensions. Higher values represent a higher quality of life [35, 36].

Before palliative care, the total score of SF-8 was 56.89 ± 21.73, with a physical sum score 56.01 ± 21.47 and a mental sum score of 57.77 ± 21.96 (Table 2, SF-8). In physical sum score, “physical functioning” had the lowest score of 48.73 ± 15.28, followed by “bodily pain” of 50.17 ± 15.37. In the mental sum score, “mental health” scored the lowest at 54.13 ± 23.23. Based on the patient-related variables (including gender, age, religion, marital status, education, occupation, medical insurance, major diagnosis, conscious state, performance status, nutritional risk, invasive experience, time from disease onset to palliative care, monthly expenses for patient) and family-related variables (including gender, age, religion, marital status, education, occupation, and relationship to patient), we found no correlation on quality of life (Table 3, SF-8).

**Table 2:** Quality-of-life, family burden, psychological impact on family members with palliative care (*N* = 230)

Item		Before PC ^a^		1 month after PC		*P*-value
		score (Mean ± SD)	*n* (%)	score (Mean ± SD)	*n* (%)	
SF-8	Total score	56.89 ± 21.73		58.15 ± 21.67		< 0.001
	Physical sum score	56.01 ± 21.47		57.10 ± 21.32		< 0.001
	General health	53.04 ± 18.88		64.35 ± 21.30		
	Physical functioning	48.73 ± 15.28		53.91 ± 19.09		
	Role-physical	56.09 ± 24.30		57.61 ± 23.87		
	Bodily pain	50.17 ± 15.37		52.52 ± 18.78		
	Mental sum score	57.77 ± 21.96		59.21 ± 21.98		< 0.001
	Vitality	57.17 ± 21.86		58.70 ± 21.68		
	Social functioning	57.72 ± 20.44		58.80 ± 21.32		
	Mental health	54.13 ± 23.23		55.76 ± 23.29		
	Role-emotional	60.37 ± 21.63		63.59 ± 20.97		
FBSD	Total score	18.86 ± 11.58		18.42 ± 10.81		< 0.001
	Economical burden	4.39 ± 3.28		4.31 ± 3.14		
	Family activities	4.55 ± 2.97		4.43 ± 2.84		
	Family entertainment	4.16 ± 2.54		4.07 ± 2.46		
	Family relationship	3.45 ± 2.74		3.34 ± 2.46		
	Physically healthy	1.20 ± 1.21		1.15 ± 1.14		
	Mental health	1.16 ± 1.20		1.12 ± 1.13		
CBI	Total score	51.84 ± 14.95		34.64 ± 20.45		< 0.001
	0–24		10 (4.35)		12 (5.22)	
	25–36		19 (8.26)		39 (16.96)	
	37–96		201 (87.39)		179 (77.83)	
HAMA	Total score	14.02 ± 11.56		13.43 ± 11.29		< 0.001
	0–6		70 (30.43)		75 (31.74)	
	7–13		58 (25.22)		62 (23.35)	
	14–20		44 (19.13)		40 (16.96)	
	21–28		35 (15.22)		33 (14.35)	
	29–56		23 (10.00)		20 (8.70)	
DT	Total score	5.23 ± 1.84		4.37 ± 1.62		0.0032
	< 5		68 (29.57)		103 (44.78)	
	≥ 5		162 (70.43)		127 (55.22)	
	Cause of distress (%) ^b^					
	Practical problem		219 (95.22)		186 (80.87)	
	Family and social problem		196 (85.22)		172 (74.78)	
	Emotional problem		211 (91.74)		103 (44.78)	
	Spiritual problem		120 (52.17)		117 (50.87)	
	Physical problem		167 (72.61)		84 (36.52)	

^a^ PC: palliative care

^b^ Values sum to more than 100% because subjects could have more than one choice

**Table 3:** Univariate regression analysis of quality-of-life, family burden, and psychologycal impact on family members before palliative care

Variable	SF-8		FBSD		CBI		HAMA		DT	
	β (95%CI)	*P*-value	β (95%CI)	*P*-value	β (95%CI)	*P*-value	β (95%CI)	*P*-value	β (95%CI)	*P*-value
**Patient-related factors**										
Gender	-0.71 (-4.79,3.38)	0.734	-1.73 (-4.91,1.45)	0.285	-2.52 (-6.62,1.58)	0.227	0.41 (-2.77,3.59)	0.798	0.05 (-0.37,0.48)	0.798
Age	-0.04 (-0.25,0.18)	0.721	0.08 (-0.09,0.25)	0.348	-0.18 (-0.4,0.03)	0.092	0.06 (-0.11,0.22)	0.506	0.01 (-0.01,0.03)	0.468
Religion	-0.45 (-9.15,8.25)	0.918	-5.51 (-12.25,1.23)	0.109	7.4 (-1.31,16.1)	0.095	3.89 (-2.86,10.64)	0.257	0.14 (-0.75,1.04)	0.751
Marital status										
Single	Ref ^a^		Ref		Ref		Ref		Ref	
Married	1.4 (-15.71,18.5)	0.872	9.16 (-4.12,22.44)	0.176	0.79 (-16.37,17.95)	0.928	-2.22 (-15.54,11.09)	0.742	0.72 (-1.04,2.48)	0.419
Divorced/ Widowed	-11.67 (-45.63,22.3)	0.499	16.33 (-10.04,42.71)	0.224	-3.67 (-37.75,30.41)	0.832	-10.33 (-36.77,16.1)	0.442	2.33 (-1.16,5.83)	0.189
Education										
≤Junior high school	Ref		Ref		Ref		Ref		Ref	
High school	0.3 (-4.29,4.89)	0.898	-0.61 (-4.17,2.96)	0.737	-3.1 (-7.68,1.49)	0.184	-0.92 (-4.41,2.57)	0.604	-0.09 (-0.56,0.38)	0.709
College	-0.57 (-5.68,4.54)	0.826	-1.09 (-5.06,2.88)	0.59	0.26 (-4.85,5.37)	0.92	4.97 (1.08,8.86)	0.012	0.06 (-0.46,0.59)	0.814
≥Graduate	-1.89 (-11.32,7.54)	0.694	-5.12 (-12.44,2.21)	0.17	4.55 (-4.86,13.97)	0.342	-3.23 (-10.41,3.94)	0.375	-0.33 (-1.3,0.64)	0.507
Occupation	1.37 (-3.42,6.17)	0.573	3.46 (-0.25,7.17)	0.067	1.84 (-2.99,6.66)	0.454	3.66 (-0.04,7.37)	0.052	-0.05 (-0.54,0.45)	0.847
Medical insurance										
Social insurance	Ref		Ref		Ref		Ref		Ref	
Commercial insurance	-1.32 (-9.99,7.34)	0.764	-0.75 (-7.54,6.03)	0.827	2.68 (-6.09,11.44)	0.548	-3.8 (-10.58,2.98)	0.27	0.2 (-0.7,1.1)	0.659
Self-paying	8.34 (-0.69,17.37)	0.07	-3.91 (-10.98,3.17)	0.278	-4.27 (-13.4,4.86)	0.358	0.06 (-7.01,7.12)	0.988	-0.11 (-1.05,0.83)	0.819
Major diagnosis										
Cancer	Ref		Ref		Ref		Ref		Ref	
Cerebrovascular disease	-0.96 (-5.85,3.93)	0.699	3.26 (-0.54,7.06)	0.092	-1.32 (-6.22,3.59)	0.597	3.5 (-0.28,7.29)	0.07	0.37 (-0.13,0.87)	0.149
Infectious disease	-3.06 (-9.07,2.95)	0.317	0 (-4.67,4.67)	0.999	3.29 (-2.74,9.32)	0.283	0.94 (-3.71,5.59)	0.691	0.51 (-0.11,1.13)	0.105
Autoimmune disease	-9.07 (-20.87,2.74)	0.132	0.17 (-9.01,9.35)	0.971	-4.95 (-16.79,6.9)	0.412	-2.17 (-11.32,6.97)	0.64	0.23 (-0.99,1.44)	0.712
Trauma	-3.92 (-14.05,6.2)	0.446	1.88 (-5.99,9.76)	0.638	-4.53 (-14.69,5.63)	0.381	-1.07 (-8.91,6.77)	0.788	0.84 (-0.2,1.88)	0.112
Conscious state										
Awake	Ref		Ref		Ref		Ref		Ref	
Somnolence	-2.03 (-7.22,3.16)	0.442	-1.03 (-5.08,3.02)	0.616	-1.43 (-6.66,3.8)	0.591	1.98 (-2.06,6.01)	0.335	-0.02 (-0.56,0.52)	0.939
Confusion	-0.61 (-5.39,4.18)	0.802	-0.05 (-3.78,3.68)	0.979	1.55 (-3.27,6.37)	0.527	-0.16 (-3.88,3.56)	0.932	0.01 (-0.48,0.51)	0.962
Coma	2.5 (-4.2,9.2)	0.463	2.55 (-2.67,7.78)	0.337	0.45 (-6.3,7.2)	0.896	-1.94 (-7.15,3.27)	0.464	0.13 (-0.57,0.82)	0.722
Performance status										
ECOG ≤ 2	Ref		Ref		Ref		Ref		Ref	
ECOG = 3	-11.93 (-29.27,5.42)	0.177	-0.43 (-13.89,13.04)	0.95	11.43 (-5.95,28.8)	0.196	-1.7 (-15.08,11.68)	0.802	-0.59 (-2.38,1.21)	0.52
ECOG = 4	-11.24 (-28.3,5.82)	0.196	-3.67 (-16.92,9.57)	0.585	7.66 (-9.43,24.76)	0.378	-5.74 (-18.9,7.42)	0.391	-0.63 (-2.4,1.13)	0.48
Nutritional risk	-9.19 (-22.59,4.21)	0.178	-2.95 (-13.37,7.47)	0.577	6.14 (-7.23,19.51)	0.366	-2.43 (-12.82,7.95)	0.645	-0.85 (-2.17,0.47)	0.208
Invasive experience	1.3 (-2.59,5.19)	0.511	3.04 (0.03,6.04)	0.048	-0.42 (-4.34,3.49)	0.831	-0.98 (-4.01,2.04)	0.524	-0.02 (-0.42,0.38)	0.921
Time ^b^ (months)	0.04 (-0.01,0.1)	0.132	-0.02 (-0.06,0.03)	0.456	-0.02 (-0.07,0.04)	0.507	0 (-0.05,0.04)	0.862	0 (-0.01,0.01)	0.898
Expense ^c^ (yuan/month)	0 (0,0)	0.887	0 (0,0)	0.187	0 (0,0)	0.308	0 (0,0)	0.944	0 (0,0)	0.431
**Family-related factors**										
Gender	0.55 (-3.32,4.43)	0.78	0.75 (-2.27,3.77)	0.625	-2.38 (-6.26,1.51)	0.23	-2.96 (-5.95,0.04)	0.053	0.28 (-0.11,0.68)	0.162
Age	-0.11 (-0.3,0.09)	0.285	0.15 (-0.01,0.3)	0.063	-0.01 (-0.21,0.19)	0.894	0.09 (-0.06,0.25)	0.231	0 (-0.03,0.02)	0.644
Religion	-8.43 (-19.63,2.78)	0.14	0 (-8.78,8.78)	0.999	-3.4 (-14.73,7.92)	0.554	4 (-4.75,12.75)	0.368	-0.93 (-2.08,0.23)	0.115
Marital status										
Single	Ref		Ref		Ref		Ref		Ref	
Married	-8.67 (-25.66,8.32)	0.316	10.1 (-3.13,23.34)	0.134	11.25 (-5.79,28.29)	0.195	7.97 (-5.22,21.17)	0.235	0.06 (-1.69,1.82)	0.942
Divorced/ Widowed	-16.67 (-37.34,4)	0.113	5.83 (-10.27,21.94)	0.476	1.33 (-19.4,22.07)	0.899	1 (-15.0565,17.0565)	0.902	-0.33 (-2.47,1.81)	0.759
Education										
High school	Ref		Ref		Ref		Ref		Ref	
College	-0.53 (-4.94,3.87)	0.812	-0.42 (-3.85,3.01)	0.812	-0.8 (-5.24,3.64)	0.724	4.83 (1.47,8.18)	0.005	0.02 (-0.44,0.47)	0.943
≥Graduate	2.1 (-3.24,7.45)	0.439	-2.37 (-6.53,1.8)	0.264	-0.38 (-5.78,5.01)	0.889	-0.24 (-4.31,3.84)	0.909	0.04 (-0.52,0.59)	0.897
Occupation										
Employed	Ref		Ref		Ref		Ref		Ref	
Unemployed	0.35 (-11.97,12.68)	0.955	-1.62 (-11.22,7.97)	0.739	1.13 (-11.34,13.59)	0.859	-4.32 (-13.93,5.29)	0.377	0.24 (-1.03,1.52)	0.708
Retirement	-3.06 (-7.03,0.92)	0.131	2.52 (-0.57,5.61)	0.11	-0.68 (-4.7,3.33)	0.738	0.84 (-2.26,3.94)	0.594	-0.08 (-0.49,0.34)	0.718
Relationship to patient										
Spouse	Ref		Ref		Ref		Ref		Ref	
Child	6.29 (-2.07,14.64)	0.139	-0.21 (-6.75,6.32)	0.949	2.14 (-6.3,10.58)	0.617	6.44 (-0.04,12.92)	0.051	0.41 (-0.45,1.28)	0.346
Sibling	11.83 (-10.39,34.04)	0.295	7 (-10.38,24.38)	0.428	8.23 (-14.22,30.68)	0.471	9.08 (-8.15,26.31)	0.3	0 (-2.3,2.3)	1

^a^Ref: reference, a value chosen as the reference value to which β were compared

^b^Time: the time from disease onset to palliative care (month)

^c^Expense: monthly expenses for patient (CNY/month)

Through palliative care, the SF-8 total score increased from 56.89 ± 21.73 to 58.15 ± 21.67, *P* < 0.001 (Table 2, SF-8). The Physical sum score was 56.01 ± 21.47 vs. 57.10 ± 21.32, *P* < 0.001, and the mental sum score was 57.77 ± 21.96 vs. 59.21 ± 21.98, *P* < 0.001.

To investigate the correlation between quality of life and palliative care, we conducted univariate and multivariate regression analyses for data associations and adjustment of covariates (Table 4, SF-8). Univariate regression analyses showed that SF-8 scores improvement were significantly related to palliative care (β 6.55, 95% CI 3.81∼9.3, *P* < 0.001). In multivariate regression analyses, various factors were adjusted in the model, including patient-related variables (gender, age, religion, marital status, education, occupation, medical insurance, major diagnosis, conscious state, performance status, nutritional risk, invasive experience, time from disease onset to palliative care, and monthly expenses for patient) and family-related variables (gender, age, religion, marital status, education, occupation, and relationship to patient). Result showed that palliative care was an independent factor affecting the quality of life of family members (β 6.55, 95% CI 3.83∼9.28, *P* < 0.001).

### Family burden

Family burden caused by patients’ diseases was tested using the Family Burden Scale of Diseases (FBSD) and Caregiver Burden Inventory (CBI) respectively before admission to palliative care and 1 month through the experience of palliative care. Higher sum scores indicate heavier family burden of diseases. Additionally, the CBI score was divided into low risk (0–24), medium risk (25–36) requiring some intervention, and high risk (> 36) at brink of “burn out” [37–39].

Before palliative care, the total score was 18.86 ± 11.58 in FBSD and 51.84 ± 14.95 in CBI (Table 2, FBSD, CBI). A total of 220 family members (95.65%) were more than medium risk and needed intervention (CBI > 24), including 201 family members (87.39%) at a high risk of “burning out” (CBI > 36). Based on the patient-related and family-related variables, there was no correlation with family burden (Table 3, FBSD, CBI).

After palliative care, the FBSD total scores decreased from 18.86 ± 11.58 to 18.42 ± 10.81, *P* < 0.001 (Table 2, FBSD), including economical burden scores of 4.39 ± 3.28 vs. 4.31 ± 3.14, family activities scores of 4.55 ± 2.97 vs. 4.43 ± 2.84, family entertainment scores of 4.16 ± 2.54 vs. 4.07 ± 2.46, family relationship scores 3.45 ± 2.74 vs. 3.34 ± 2.46, physically health scores of 1.20 ± 1.21 vs. 1.15 ± 1.14, and mental health scores of 1.16 ± 1.20 vs. 1.12 ± 1.13. CBI scores decreased from 51.84 ± 14.95 to 34.64 ± 20.45, *P* < 0.001 (Table 2, CBI). The proportion of low risk (0–24) within the CBI score increased from 4.35 to 5.22%, with medium risk increasing from 8.26% to 16.96, and high risk significantly decreasing from 87.39 to 77.83%.

Univariate and multivariate regression analyses were conducted for data associations and adjustment of covariates in family burden (Table 4, FBSD and CBI). Univariate regression analyses showed that FBSD scores (β 0.47, 95% CI -5.52∼-1.58, *P* = 0.032) and CBI scores (β -5.24, 95% CI -7.85∼-2.63, *P* < 0.001) both decreased significantly with palliative care. In multivariate regression analyses, covariates were adjusted for both patient-related and family-related variables. Results showed that palliative care was an independent factor affecting the family burden of family members (β -0.48, 95% CI -2.50∼-1.54, *P* = 0.0341 in FBSD; β -5.24, 95% CI -7.84∼-2.65, *P* < 0.001 in CBI).

### Psychological impact

The psychological impact of the family members was tested using the Hamilton Anxiety Scale (HAMA) and Distress Thermometer (DT) respectively before admission to palliative care and 1 month through the experience of palliative care. Higher sum scores indicate heavier psychological burden from patients and diseases. HAMA score was divided into no anxiety (≤ 7), possible anxiety (7–14), definitely anxiety (14–21), obvious anxiety (21–29), and severe anxiety levels (≥ 29) [40, 41]. Additionally, in DT, a cut-off value of ≥ 5 is considered clinically relevant distress with a need of professional psychological support [42, 43].

Before palliative care, high levels of psychological impact were shown in both HAMA and DT. With regards to HAMA, 102 family members (44.35%) were rated as having anxiety, with 44 as definitely anxiety (19.13%), 35 as obvious anxiety (15.22%), and 23 as severe anxiety (10.00%). The total scores of HAMA were 14.02 ± 11.56, which were in the definitely anxiety range (Table 2, HAMA). In terms of the DT, clinically relevant distress (≥ 5) was in 162 of 230 family members (70.43%). The total score of DT was 5.23 ± 1.84. Out of five types of problems on the list, practical problem (95.22%), emotional problem (91.74%), and family and social problem (85.22%) were the three most serious issues indicated by family members (Table 2, DT). Both patient-related and family-related variables had no impact on psychological outcomes (Table 3, HAMA, DT).

After palliative care, HAMA total score decreased from 14.02 ± 11.56 to 13.43 ± 11.29, *P* < 0.001 (Table 2, HAMA). Different levels anxiety (> 14) proportions decreased from 44.35 to 40.01% (*P* < 0.05), with definitely anxiety (14–20) decreasing from 19.13 to 16.96%, obvious anxiety (21–28) from 15.22 to 14.35%, and severe anxiety (≥ 29) from 10.00 to 8.70%. DT total score decreased from 5.23 ± 1.84 to 4.37 ± 2.62, *P* < 0.01 (Table 2, DT). The proportion of clinically relevant distress (> 5) decreased from 70.43 to 55.22%. The focus is still practical problems (80.87%). The most obvious reduction was in emotional problems (91.74–44.78%, *P* < 0.05), followed by physical problems (72.61–36.52%, *P* < 0.05), and practical problems (95.22% vs. 80.87%, *P* < 0.05).

Univariate and multivariate regression analyses were conducted for data associations and adjustment of covariates in family burden (Table 4, HAMA and DT). Univariate regression analyses showed that the decrease in HAMA scores (β -0.68, 95% CI -2.77∼-1.41, *P* = 0.0422) and DT scores (β -0.1, 95% CI -0.37∼-0.18, *P* = 0.0498) was associated with palliative care. In multivariate regression analyses, covariates were adjusted for both patient-related and family-related variables. The results showed that palliative care was an independent factor affecting the psychological impact of family members (β -0.68, 95% CI -2.69∼-1.32, *P* = 0.0405 in HAMA; β -0.1, 95% CI -0.37∼-0.18, *P* = 0.0496 in DT).

**Table 4:** Univariate and multivariate regression analysis of quality-of-life, family burden, and psychological impact on family members with palliative care (*N* = 230)

Item	Variable	Univariate Model		Multivariate-Adjusted Model ^a^	
		β (95% CI)	*P*-value	β (95% CI)	*P*-value
SF-8	Before PC ^b^	Ref ^c^		Ref	
	After PC	6.55 (3.81 ~ 9.3)	< 0.001	6.55 (3.83 ~ 9.28)	< 0.001
FBSD	Before PC	Ref		Ref	
	After PC	-0.47 (-2.52~-1.58)	0.032	-0.48 (-2.50~-1.54)	0.0341
CBI	Before PC	Ref		Ref	
	After PC	-5.24 (-7.85~-2.63)	< 0.001	-5.24 (-7.84~-2.65)	< 0.001
HAMA	Before PC	Ref		Ref	
	After PC	-0.68 (-2.77~-1.41)	0.0422	-0.68 (-2.69~-1.32)	0.0405
DT	Before PC	Ref		Ref	
	After PC	-0.1 (-0.37~-0.18)	0.0498	-0.1 (-0.37~-0.18)	0.0496

^a^ Multivariate-Adjusted Model: adjusted for patient-related variables, including gender, age, religion, marital status, education, occupation, medical insurance, major diagnosis, conscious state, performance status, nutritional risk, invasive experience, time from disease onset to palliative care, and monthly expenses for patient, and family-related variables, including gender, age, religion, marital status, education, occupation, and relationship to patient

^b^ PC: palliative care

^c^ Ref: a value chosen as the reference value to which β were compared

## Discussion

This prospective study investigated the influence of palliative care on quality-of-life, family burden, and psychological impact through a series of scales, and finally demonstrates the availability and stability of palliative care for family members of terminally ill patients in the health and social care integrated institution.

Palliative care regards death as a normal part of life that neither accelerates nor delays the process. It provides active services to terminal ill patients to make life as comfortable and dignified as possible while providing assistance to families. Effective palliative care works for both patients and their families through early identification, assessment, and treatment of physical, psychological, social, and spiritual symptoms and needs and supports them in quality of life, family burden, and psychological status [44–46].

Previous studies demonstrated that quality of life in family caregivers (FCs) of newly diagnosed patients with incurable cancer was lower than normalized mean in the U.S. population [47], while findings indicated the result to be associated with their relationship to the patients [48, 49]. In a recent study, Anneke Ullrich, et al. reported that many FCs suffer from relevant impaired quality of life and decreased psychosocial well-being during specialized inpatient palliative care (SIPC), with no correlation with the relationship to the patients in their study [24]. FCs’ quality of life was considered to be influenced by the quality of patient care [24, 50, 51]. This effectiveness might be biased by social desirability during the patients’ stay on the SIPC ward and the dependency on inpatient palliative care. Similar findings were obtained in our study, which is that palliative care effectively improved quality of life of family members, reduced family burden, and relieved psychological impact.

Although previous studies have explored the preference of terminally ill patients, palliative care studies have mostly focused on oncology, and intensive care units (ICU) [52–55] and have not addressed patients in the final stages of all diseases and the inevitable end-of-life problems of all elderly patients [56–58]. The integration of health and social care for elder adults, as a means to solve the aging of society, facing multiple sources of terminally ill elderly people, is more consistent with the social aging model [59, 60].Therefore, this study was the first to examine the effectiveness of palliative care at the integrated institution for health and social care, and 230 eligible family members of patients with terminal illnesses were enrolled in the study. Compared to hospital specialist wards or community palliative care, this study possesses the characteristics of stable follow-up and reliable observation results and has more advantages in information collection, data analysis, and adjustment of confounding [61, 62].

In our study, we assessed several key components related to the well-being of family members of terminally ill patients receiving palliative care, including quality of life, family burden, and psychosocial status applied corresponding scales [36–43]. These components serve as crucial indicators of the effectiveness of palliative care interventions and have important implications for both practice and research in the field of palliative care. The scale was primarily developed and validated in cancer patients, but also has been found to be a suitable tool for measuring family members of patients receiving palliative care [63, 64]. Most family members are under extreme pressure in quality of life, family burden, and psychosocial status. The significant changes observed in scale results following palliative care implementation suggest a potential association between the intervention and the observed outcomes. Our findings demonstrate the positive impact of palliative care on the quality of life of family members, highlighting the importance of integrating comprehensive palliative care programs that address the emotional, social, and spiritual aspects of care. Palliative care interventions were associated with a reduction in family burden and improvements in psychosocial status among family members, underscoring the need for tailored support services to mitigate practical, financial, and emotional challenges. However, It should be noted that while the changes in indicators before and after palliative care are statistically significant, some indicators, especially FBSD, do not show substantial changes in specific score values. This may be due to the large sample size, where even minor score differences can achieve statistical significance. Therefore, it is essential to analyze these findings in practice by considering the clinical significance of different assessment scales.

Additionally, we took the advantage of the integrated institution for health and social care to record extensive covariables and conduct research observations as long as possible. Multivariate regression analysis determined the relationship between palliative care and the positive impact of family members on quality of life, family burden, and psychosocial status, with patient-related and family-related adjusted models. The association was independent of patient-related and family-related factors, which proved that the results are stable and widely applicable.

Moving forward, research should aim to further understand the specific components of palliative care that contribute most significantly to improvements in family well-being, conduct longitudinal studies to examine the long-term effects of palliative care interventions, and compare the effectiveness of different palliative care models to optimize outcomes for families facing terminal illness.

## Strengths and limitations

The strengths of this study are its population and setting range. We selected all the patients with terminal illnesses and their members at the integrated institution for health and social care for the first time. The follow-up and observation are stable, and the demographic data are collected more comprehensively.

However, there are several limitations to consider. Firstly, although we adjusted for as many patient-related and family-related covariates as possible, as in all observational studies, there may have been still some potential uncontrolled confounders. Secondly, our study was only conducted on one of the regions with the program (integrated institution for health and social care) without a control group for measures for the individuals before palliative care. Furthermore, as a new way to cope with the aging of society, integrated institutions for health and social care have only been established for a few years. The sample size of our study on palliative care in the integrated institution health and social care is limited. Our investigation will continue to be conducted, with the development of the institutions and the passage of time. Additionally, we acknowledge the limitations of the SF-8 scale used to assess quality of life in this study, which primarily focuses on physical functioning wtihout fully capture the multidimensional nature of quality of life experienced by participants. We recognize the need for more comprehensive measurement tools and plan to supplement them in future research endeavors.

## Conclusion

In conclusion, family members of terminally ill patients suffer from low quality of life, heavy family burden, and severe psychological impact, with no effect of both patient-related and family-related factors. In the integrated institution for health and social care, palliative care can effectively improve quality of life, reduce the family burden, and relieve the psychological impact on family members of terminally ill patients. In addition, palliative care was an independent influencing factor on quality of life, family burden, and psychosocial status. Independent of patient-related and family-related factors, the results are stable and widely applicable. These results demonstrate the availability and stability of palliative care for family members with advanced illness patients in the integrated institution for health and social care. Our findings underline the importance of palliative care and the popularization of the service model of health and social care integration for elderly adults.

## Data Availability

The original contributions presented in this study are included in the article materials, further inquiries can be directed to the corresponding author.
